# Characterization of Formononetin Sulfonation in SULT1A3 Overexpressing HKE293 Cells: Involvement of Multidrug Resistance-Associated Protein 4 in Excretion of Sulfate

**DOI:** 10.3389/fphar.2020.614756

**Published:** 2021-01-11

**Authors:** Fanye Liu, Shuhua Pei, Wenqi Li, Xiao Wang, Chao Liang, Ruohan Yang, Zhansheng Zhang, Xin Yao, Dong Fang, Songqiang Xie, Hua Sun

**Affiliations:** ^1^ Institute for Innovative Drug Design and Evaluation, School of Pharmacy, Henan University, Kaifeng, China; ^2^ Institute of Chemical Biology, School of Pharmacy, Henan University, Kaifeng, China

**Keywords:** formononetin, sulfonation, efflux transporter, MRP4, HEK293 cells

## Abstract

Formononetin is one of the main active compounds of traditional Chinese herbal medicine *Astragalus membranaceus*. However, disposition of formononetin via sulfonation pathway remains undefined. Here, expression-activity correlation was performed to identify the contributing of SULT1A3 to formononetin metabolism. Then the sulfonation of formononetin and excretion of its sulfate were investigated in SULT1A3 overexpressing human embryonic kidney 293 cells (or HKE-SULT1A3 cells) with significant expression of breast cancer resistance protein (BCRP) and multidrug resistance-associated protein 4 (MRP4). As a result, formononetin sulfonation was significantly correlated with SULT1A3 protein levels (r = 0.728; *p* < 0.05) in a bank of individual human intestine S9 fractions (n = 9). HEK-SULT1A3 cells catalyzed formononetin formation of a monosulfate metabolite. Sulfate formation of formononetin in HEK-SULT1A3 cell lysate followed the Michaelis-Menten kinetics (V_max_ = 13.94 pmol/min/mg and K_m_ = 6.17 μM). Reduced activity of MRP4 by MK-571 caused significant decrease in the excretion rate (79.1%–94.6%) and efflux clearance (85.3%–98.0%) of formononetin sulfate, whereas the BCRP specific inhibitor Ko143 had no effect. Furthermore, silencing of MRP4 led to obvious decrease in sulfate excretion rates (>32.8%) and efflux clearance (>50.6%). It was worth noting that the fraction of dose metabolized (f_met_), an indicator of the extent of drug sulfonation, was also decreased (maximal 26.7%) with the knockdown of MRP4. In conclusion, SULT1A3 was of great significance in determining sulfonation of formononetin. HEK-SULT1A3 cells catalyzed formononetin formation of a monosulfate. MRP4 mainly contributed to cellular excretion of formononetin sulfate and further mediated the intracellular sulfonation of formononetin.

## Introduction

Traditional Chinese Medicine (TCM) has a history of more than 2000 years in diagnosis and treatment of diseases. In recent years, more and more research has been devoted to the study of TCM, including their pharmacological activities, effective forms, and mechanisms of action. *Astragalus membranaceus* (Huangqi), one of the most well-known TCM in China and other oriental countries, is widely used as an important component in many Chinese medicine formulas. In more than 2000 years of clinical practice and pharmacological research, *Astragalus membranaceus* has shown multiple effects, such as protection of cardiovascular system (Liu D. et al., 2018), liver-protective functions (Mohibbullah et al., 2019), antioxidant (Shanzad et al., 2016), antidiabetes (Li et al., 2019a), immunomodulatory (Li et al., 2020), and antitumor (Liu et al., 2019; Cheon and Ko, 2020) activities. It is worth noting that the phase 1 dose-escalation study of a new herbal medicine SH003, containing Huangqi (*Astragalus membranaceus*), has been finished in patients with solid cancers. Due to extensive research, triterpene saponins, flavonoids, polysaccharides, and phenolic acids were identified as the main active components (Li et al., 2019b; Wang et al., 2019).

Formononetin is one of the most investigated ingredients in *Astragalus membranaceus* (Huangqi) (Zheng et al., 2019). Formononetin has been under intense investigation for the past decade and numerous biological activities have been reported. Strong evidence has shown that formononetin can be used as an anticancer agent against diverse cancers through promoting apoptosis, working against proliferation, and inhibiting metastasis of cancer cells *in vitro* and *in vivo* (Ong et al., 2019). Also, formononetin's antioxidant and neuroprotective effects suggest its potential for Alzheimer's disease treatment (Sun et al., 2012; Xiao et al., 2019). Further, formononetin can effectively inhibit atherosclerosis, thus showing protective effect on cardiovascular system (Ma et al., 2020).

Till now, extensive research has focused on pharmacology activities of formononetin. Several studies have investigated the pharmacokinetic of formononetin in human, rats, or mice after oral administration of individual compound, Chinese patent medicine, herbal extracts of *Astragalus membranaceus*, or Chinese herbal formulas (Guan et al., 2014; Guo et al., 2015; Liu et al., 2015; Luo et al., 2018; Rao et al., 2019). However, the metabolic pathway of formononetin *in vivo or in vitro* is not reported. Formononetin is a member of the class of 7-hydroxy isoflavones according to its chemical structure. It was known that flavonoids could be rapidly metabolized to form their phase II metabolites (such as glucuronides and sulfates), especially in intestine, thus limiting their oral absorption, resulting in exceedingly low exposure (Chen et al., 2014; Chalet et al., 2018; Ravisankar et al., 2019). Further, inhibition of phase II metabolism could effectively increase bioavailability of flavonoids (Zhang et al., 2016; Ravisankar et al., 2019). Therefore, to clarify the disposition of formononetin *in vivo* is very necessary, especially phase II metabolism.

It has been certified that the plasma concentration of formononetin glucuronide in rats, generated by UDP-glucuronosyltransferase (UGTs) after oral administration, was much higher than the parent compound (Shi et al., 2015). However, the sulfonation of formononetin was rarely reported. Sulfonation is another important phase II metabolism mediated by sulfotransferase (SULTs), in addition to glucuronidation. Moreover, several researches have revealed that ATP-binding cassette (ABC) transporters, such as breast cancer resistance protein (BCRP) and multidrug resistance-associated proteins (MRPs), mediated the transmembrane transport of glucuronides and sulfates of flavonoids (Sun et al., 2015; Liu et al., 2018; Qi et al., 2019). Inhibition of the effect of efflux transporters on metabolites excretion could significantly decrease the cellular glucuronidation or sulfonation (Zhang et al., 2015; Liu et al., 2018). However, the effects of efflux transporters on formononetin sulfate excretion or sulfonation have not been determined.

Here, we have investigated sulfonation disposition of formononetin using HEK-SULT1A3 cells model in this study. SULT1A3 was appropriately selected because it is exclusively sulfated at 7-hydroxy (7-OH) position of flavonoids (Meng et al., 2012). Also, SULT1A3 was one of the most expressed SULT family isoforms in human intestine which indicated that it may play an important role in flavonoids disposition *in vivo* (Riches et al., 2009). Furthermore, the contributions of efflux transporters (i.e., BCRP and MRPs) to formononetin sulfate excretion were determined by using combined approaches of chemical inhibition (Ko143 for BCRP and MK-571 for MRPs) and biological inhibition (shRNA-mediated gene silencing).

## Materials and Methods

### Chemicals and Reagents

Recombinant human SULT1A3 enzymes (rSULT1A3) were purchased form Sekisui XenoTech LCC (Kansas, USA). Formononetin (purity >98%) was purchased from Baoji Herbest Bio-Tech Co., Ltd. (Baoji, China). Acetonitrile, methanol, and water, which were of LC-MS grade, were bought from Merk KGaA (Darmstadt, Germany).

### Sulfonation Assay

Enzyme kinetic of formononetin incubated with recombinant human SULT1A3 enzyme (rSULT1A3) was evaluated. The reaction was activated after the coenzyme PAPS (which provides sulfonate group to the substrates) was added. The incubation reaction was conducted in 200 μL potassium phosphate buffer (50 mM, pH 7.4) at 37°C, containing SULT1A3 enzyme (0.1 mg/ml), formononetin (0.16–40 μM), and PAPS (a final concentration of 0.10 mM). After incubation for 120 min, the reaction was terminated by adding 100 μL ice-cold acetonitrile. Then the samples were vortexed and centrifuged at 15,000 g for 15 min. The supernatant was analyzed by HPLC for sulfate quantification. All incubations were performed in triplicate. Preliminary experiments were performed to ensure that the rates of sulfation were determined under linear conditions with respect to incubation time and protein concentration.

### Kinetic Evaluation

The sulfonation rates of formononetin at different concentrations were determined according to the sulfonation assay protocol. The kinetic model Michaelis-Menten (Eq. 1) was fitted to the data of sulfonation rates vs. formononetin concentrations. Model selection was based on visual inspection of the Eadie-Hofstee plot. Linearization of the Eadie-Hofstee plots indicated that the data were best described by Michaelis-Menten model. Model fitting and kinetic parameters evaluation were performed using GraphPad Prism software (5.3 version).

$$V=\frac{V_{max}\cdot \left[S\right]}{K_{m}+\left[S\right]}$$ (1)

where V_max_ is the maximal velocity and K_m_ is the Michaelis constant. The intrinsic clearance (CL_int_) was calculated by V_max_/K_m_.

### Structure Identification of Formononetin Sulfate by UPLC-QTOF/MS

The structure of formononetin sulfate was identified using UPLC-QTOF/MS method. Briefly, samples were separated using the Waters ACQUITY UPLC system and BEH column (2.1 × 50 mm, 1.7 μm; Waters, Milford, MA). Gradient elution was performed using formic acid (0.1%) in water (mobile phase A) and acetonitrile (mobile phase B) at a flow rate of 0.45 ml/min. Elution condition was 10% B at 0–1.0 min, 10%–90% B at 1.0–3.0 min, 90% B at 3.0–3.5 min, and 90%–10% B at 3.5–4.0 min. After chromatographic separation, samples were analyzed through Xevo G2 QTOF/MS (Waters) in the negative mode. The precursor and fragment ion information was collected using the electrospray ionization source (ESI) in MSMS mode. The collision energy was 15 eV. Data were analyzed using MassLynx version 4.1.

### Quantification of Formononetin Sulfate by HPLC Analyses

Quantification of formononetin sulfate was performed using Agilent 1,260 Infinity II HPLC system with a ZORBAX SB-C18 column (4.6 × 250 mm, 5 μm; Agilent). The temperature of the column was set at 40 °C. The samples were analyzed using a gradient of ammonium acetate (2.5 mM) in water (mobile phase A) vs. acetonitrile (mobile phase B) at a flow rate of 1.0 ml/min. The gradient elution condition was 15% B at 0–2.0 min, 15–95% B at 2.0–12 min, 95% B at 12–15 min, and 95–15% B at 15–18 min. The waiting time was 7.0 min at 15% B in order to ensure the column pressure is back to a stable state before the next injection. The injection volume was 25 μL and the detection wavelength of formononetin sulfate was 250 nm. The standard curve was conducted using formononetin sulfate purified by our laboratory.

### Correlation Analysis

Expression-activity correlation was performed to identify the role of SULT1A3 enzyme in formononetin sulfonation. The protein expression of SULT1A3 in individual human intestine S9 fractions (n = 9) was evaluated using western blotting assay. The sulfonation activities of nine individual human intestine S9 fractions toward formononetin were determined according to the sulfonation assay protocol. Correlation (Pearson) analyses were performed between protein expression levels of SULT1A3 enzyme and formononetin sulfonation in nine individual human intestine S9 fractions by GraphPad Prism software (5.3 version).

### Functional Identification of SULT1A3 Overexpressing HKE293 Cells

HEK293 cells were stably transfected with the cDNA of Human SULT1A3 enzyme through the lentiviral transduction approach (Sun et al., 2015), which were named as HEK-SULT1A3 cells. The transduction efficiency was evaluated by measuring the SULT1A3 protein levels in both control and transfected cells. The enzyme function of HEK-SULT1A3 cells was further determined using sulfonation assay. In brief, HEK-SULT1A3 cell lysate was collected. Then, in order to identify the function of HEK-SULT1A3 cells in sulfate generation, formononetin was incubated with the HEK-SULT1A3 cell lysate and enzyme kinetic was determined as described above.

### Sulfate Excretion Experiments

The sulfate excretion experiments were conducted using HEK-SULT1A3 cells model. In brief, HEK-SULT1A3 cells were washed with Hank's Balanced Salt Solution (HBSS) buffer (37°C, pH = 7.2) for three times. Then the cells were incubated with 2 ml HBSS buffer containing different concentrations of formononetin (final concentrations of 2.5 μM or 10 μM) at 37°C. 200 μL of incubation medium was sampled from each well at various times (0.5, 1.0, 1.5, and 2.0 h). Meanwhile, 200 μL of dosing solution was added immediately. The extracted samples were mixed with 100 μL ice-cold acetonitrile and vortexed for 1 min. The supernatant after centrifugation (15,000 g; 15 min) was injected into HPLC for analysis. After the last samples were taken, cells were collected into 400 μL ice-cold MeOH:H_2_O (1:1, v/v) and processed to measure the intracellular amount of formononetin sulfate. The excretion rate (ER) of intracellular sulfate was calculated exactly as described in our publications (Liu et al., 2018). The apparent efflux clearance (CL_ef,app_) was derived as the excretion rate divided by the intracellular concentration of sulfate (Ci). Ci was determined by dividing intracellular amount of sulfate at 2 h by intracellular volume. The intracellular volume of HEK293 cells was measured using sulfanilamide as described (Zhou et al., 2015).

### Effect of Chemical Inhibitor on Sulfate Excretion

For this study, Ko143 (the specific inhibitor of BCRP) or MK-571 (the span inhibitor of MRPs) was used to inhibit the efflux transport function of BCRP or MRP4, and then the excretion rate and apparent efflux clearance (CL_ef,app_) of formononetin sulfate in HEK-SULT1A3 cells were evaluated as described above.

### Transient Transfection of Short-Hairpin RNA Targeting BCRP or MRP4

The shRNA targeting MRP4 (NM_005845) was subcloned to the pLKO.1-Puro vector. Then the recombinant plasmid or blank vector (scramble) was transfected into HEK-SULT1A3 cells using Lipofectamine 2000 reagent (Invitrogen, Waltham, MA). Cells were used for sulfate excretion experiments 48 h after transfection. Further, the fraction of dose metabolized (f_met_), an indicator of the extent of drug sulfonation, was calculated according to Eq. 2.

$$f_{met}=\frac{\text{excreted sulfate}+\text{intracellular sulfate}}{\text{total dosed aglycone}}$$ (2)

### Western Blotting Analysis

The cells were collected and lysed in RIPA Lysis Buffer (Beyotime, Shanghai) in advance. After quantified using BCA protein assay kit (Thermo Scientific, Waltham, Massachusetts), the cells samples or individual human intestine S9 fractions were analyzed by sodium dodecyl sulfate polyacrylamide gel electrophoresis (10% acrylamide gels) and transferred to polyvinylidene fluoride membranes (0.22 μM, Millipore, Bedford, MA). After blocking for 1 h, the membranes were incubated with primary antibodies (1:1,000 dilution) at 4°C overnight, followed by anti-rabbit or anti-mouse horseradish peroxidase conjugated secondary antibody. Target protein bands were visualized with enhanced chemiluminescence and imaged by autoradiography. And then the protein bands were scanned in grayscale and subjected to semiquantitative analysis using Quantity One software.

### Statistical Analysis

Data were presented as mean ± standard deviation (SD). Statistically significant differences between two group data were analyzed by two-tailed Student’s t-test. The level of significance was set at *p* < 0.05.

## Results

### Structure Identification of Formononetin Sulfate

**FIGURE 1:**
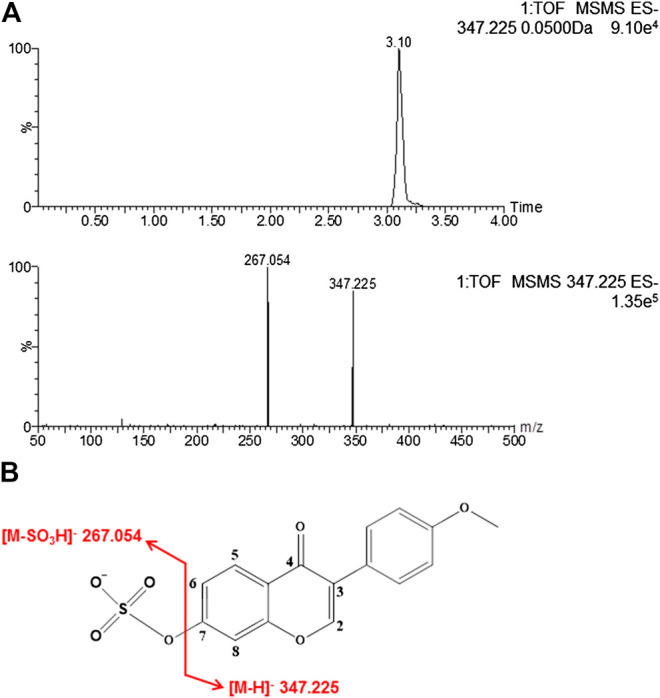
The chromatogram of formononetin sulfate under MS/MS scanning **(A)** and the fragmentation pathways of formononetin sulfate **(B)**.

The retention time of formononetin sulfate was 3.10 min (Figure 1A). Formononetin sulfate formed a deprotonated molecule [M-H]^-^ at m/z 347.225 in the negative ion scan mode. The major fragment ion of the sulfate was observed at m/z 267.054, which corresponded exactly to the parent compound (formononetin). The [M-H]^-^ mass of formononetin sulfate showed an exact mass difference of a sulfonate group compared to the parent compound, indicating that this metabolite was a monosulfate of formononetin (Figure 1A,B).

### Generation of Formononetin Sulfate in rSULT1A3 and HEK-SULT1A3 Cell Lysate

**FIGURE 2:**
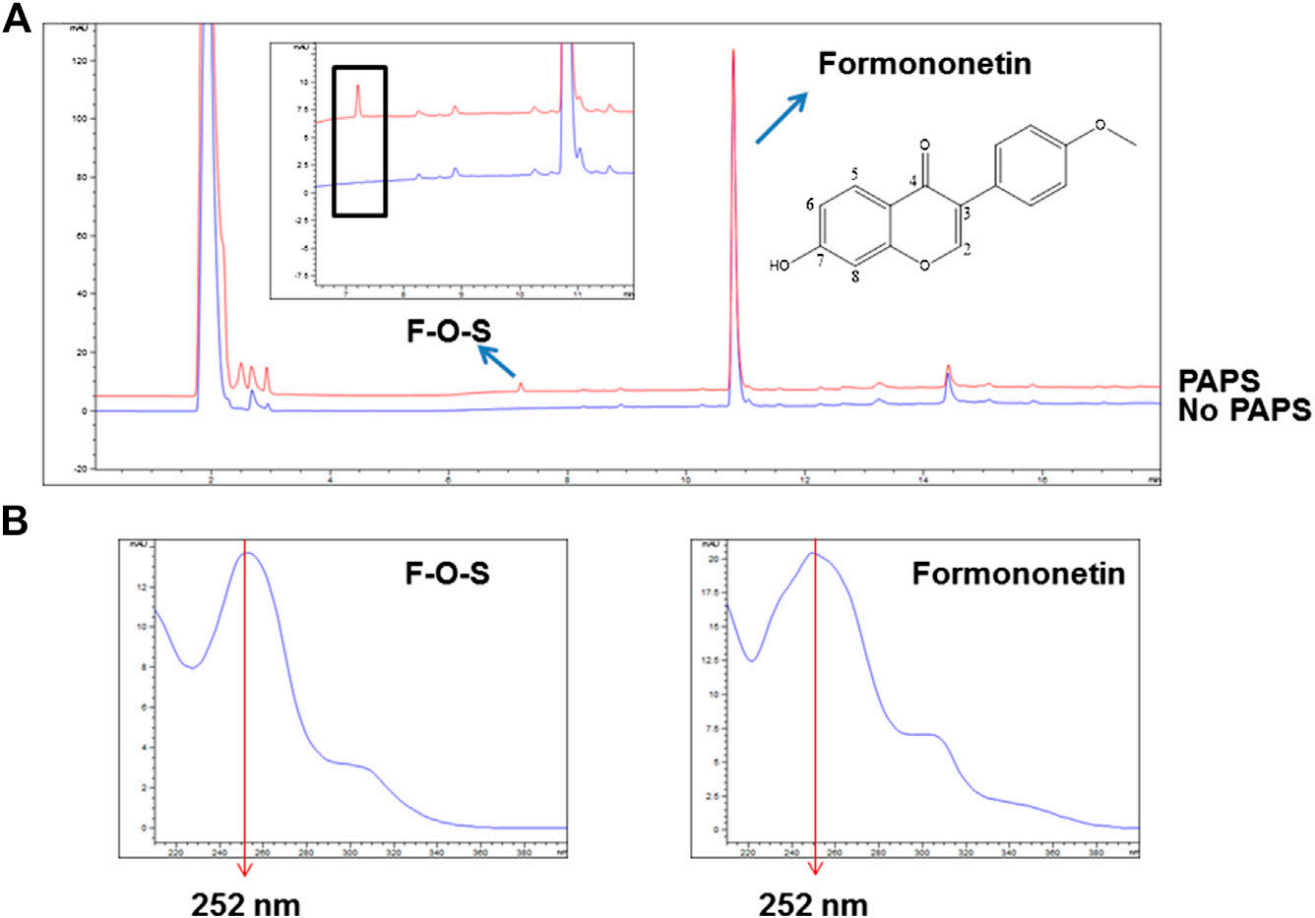
**(A)** Chemical structure of formononetin and representative HPLC chromatograms for quantitative analyses of formononetin and its sulfate (F-O-S). **(B)** UV absorption spectra of formononetin and F-O-S.

3′-Phosphoadenosine-5′-phosphosulfate (PAPS) was necessary for sulfonation reaction catalyzed by sulfotransferases. When incubating formononetin with rSULT1A3 enzyme, one formononetin sulfate (F-O-S) was generated in the presence of PAPS (Figure 2A). By contrast, no sulfate formed in the absence of PAPS under the same incubation condition (Figure 2A). Under the effect of sulfotransferases, a sulfonate group was transformed form PAPS to the 7-hydroxy of formononetin. As a result, the water solubility of metabolite was enhanced compared to parent compound formononetin. Accordingly, the metabolite was eluted much earlier (7.25 min) compared with formononetin (10.79 min) (Figure 2A). In addition, the metabolite showed high similarity of UV absorption spectrum (*λ*_max_ = 252 nm) with that of parent compound (Figure 2B).

**FIGURE 3:**
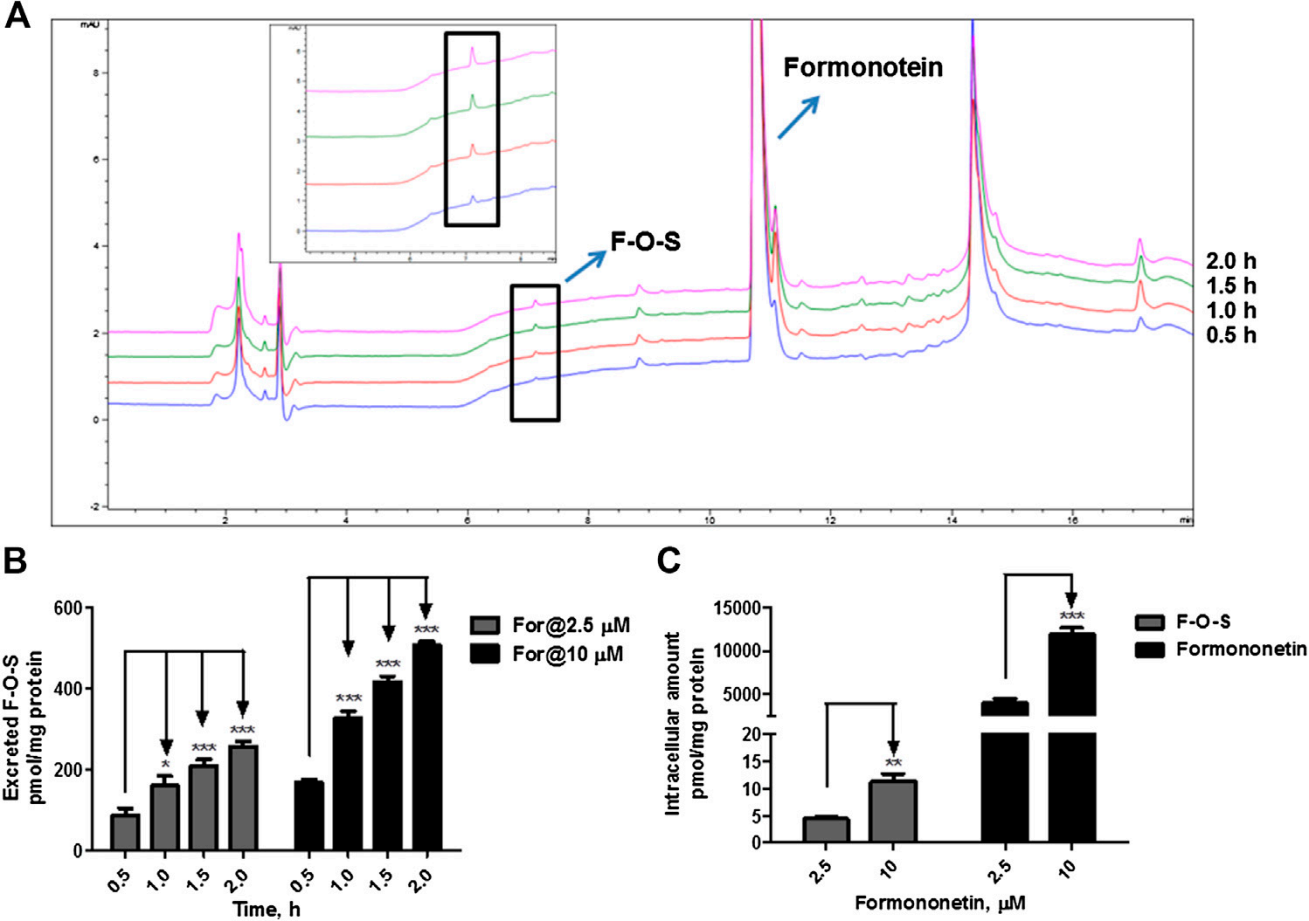
Disposition of formononetin in HEK-SULT1A3 cells at different loading doses. **(A)** Representative HPLC chromatograms showing that formononetin sulfate (F-O-S) was generated after incubation with HEK-SULT1A3 cells at different time points and excreted into extracellular medium. **(B)** Excretion rates of formononetin sulfate (F-O-S) at dose of 2.5 μM and 10 μM, respectively. **(C)** Intracellular amounts of formononetin and F-O-S at 2 h after incubation under different loading doses. *p < 0.05, **p < 0.01, ***p < 0.001 compared with control. Each data point was shown as mean ± SD (n = 3).

When formononetin (2.5 μM or 10 μM) was incubated with HEK-SULT1A3 cells, F-O-S was generated and excreted into the extracellular media. The amount of metabolite increased with the incubation time (Figure 3A). Further, the extracellular amounts of F-O-S were significantly increased (*p* < 0.001) as the dose increased from 2.5 to 10 μM (Figure 3B). Increased sulfate excretion was associated with an elevated level of formononetin within the cells (Figure 3C). The above results indicated that HEK-SULT1A3 cells were a suitable model to investigate the disposition of drugs and excretion of their sulfate metabolites.

### Activity-Expression Correlation

**FIGURE 4:**
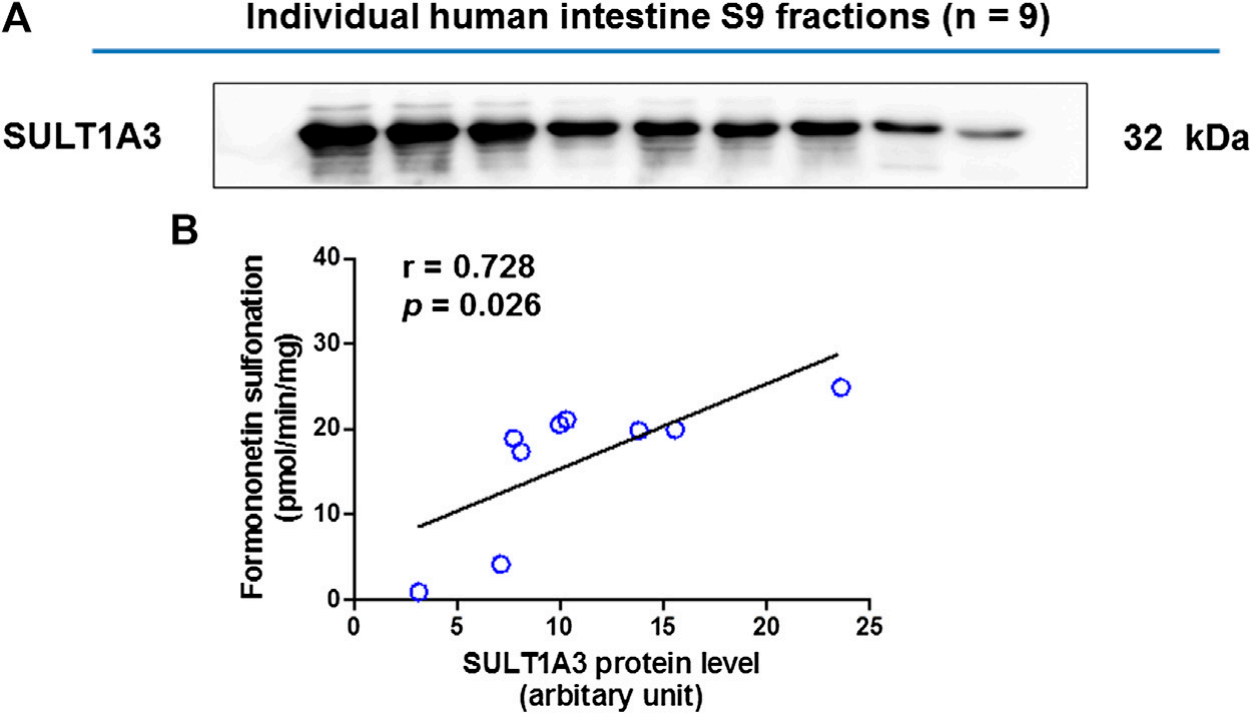
**(A)** Protein expression of SULT1A3 in a bank of individual human intestine S9 fractions (n = 9). **(B)** Correlation analysis between formononetin sulfonation and SULT1A3 protein levels in individual human intestine S9 fractions (n = 9).

The SULT1A3 levels in a bank of individual human intestine S9 fractions (n = 9) were quantified using the immunoblotting technique (Figure4A). The sulfonation activities of these nine individual human intestine S9 fractions toward formononetin (5 μM) were measured. It was found that formononetin sulfonation velocity was significantly correlated with the protein levels of SULT1A3 (r = 0.728; *p* < 0.05) (Figure 4B). These results indicated that SULT1A3 was of great significance in determining sulfonation of formononetin.

### Enzyme Kinetics of Formononetin in rSULT1A3 and HEK-SULT1A3 Cell Lysate

**FIGURE 5:**
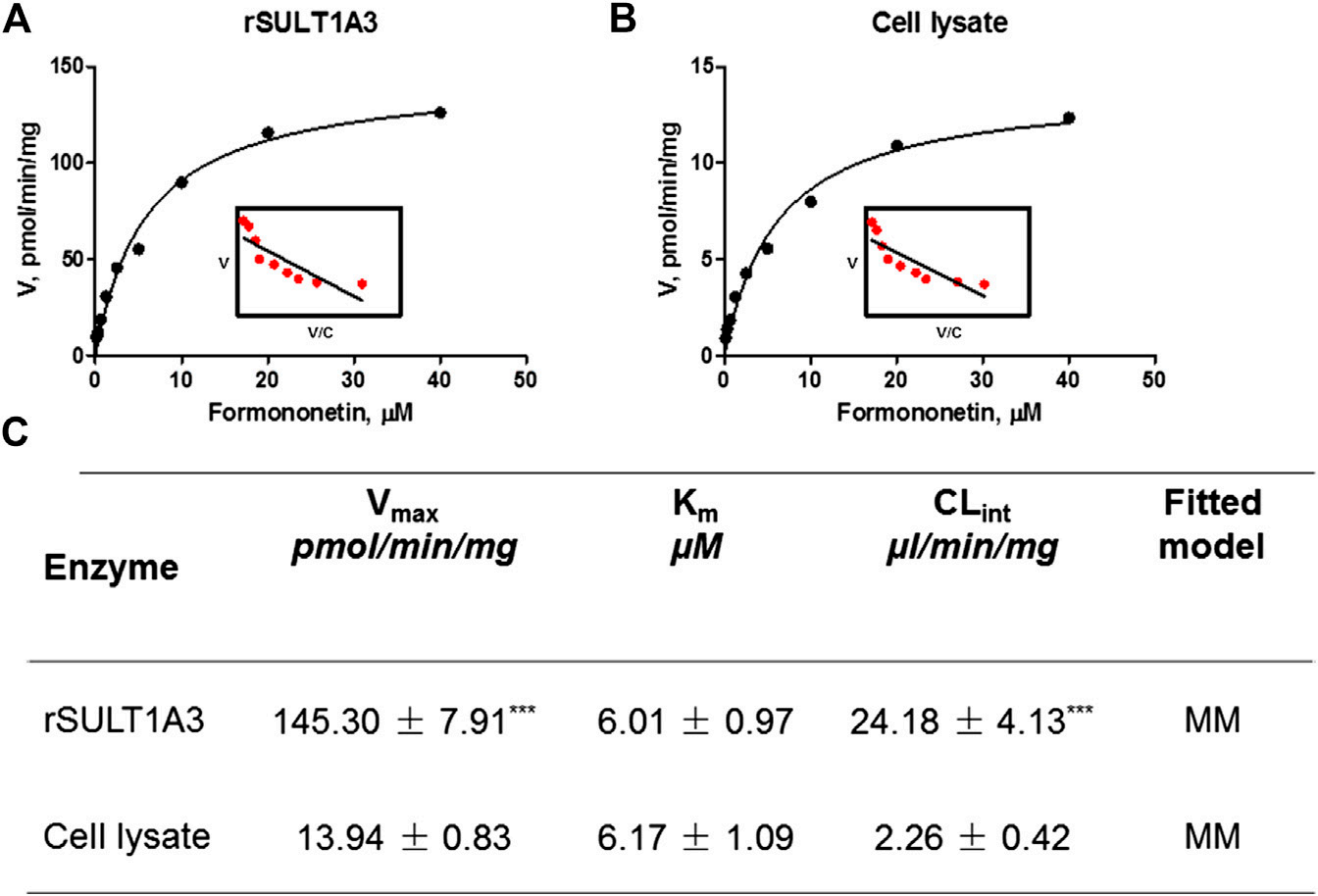
**(A)** Enzyme kinetic profile and Eadie-Hofstee plot (insert) of formononetin sulfonation in rSULT1A3. **(B)** Enzyme kinetic profile and Eadie-Hofstee plot (insert) of formononetin sulfonation in HEK-SULT1A3 cell lysate. **(C)** Kinetic parameters derived for formononetin sulfonation in rSULT1A3 enzyme and HEK-SULT1A3 cells lysate. Each data point was shown as mean ± SD (n = 3). ***p < 0.001 compared with cell lysate. Each data point was shown as mean ± SD (n = 3).

As shown in Figure 5A, B, formation of F-O-S in rSULT1A3 and HEK-SULT1A3 cell lysate both followed the classical Michaelis-Menten kinetics. The value of V_max_ was 145.30 ± 7.91 pmol/min/mg and 13.94 ± 0.83 pmol/min/mg for formononetin in rSULT1A3 and cell lysate, respectively. The value of K_m_ was 6.01 ± 0.97 μM and 6.17 ± 1.09 μM in rSULT1A3 and cell lysate, respectively. Because the protein purity of SULT1A3 enzyme was different, the V_max_ of formononetin sulfonation was much lower (*p* < 0.001) in cell lysate than that in rSULT1A3 enzyme (Figure 5C). However, the K_m_ value in cell lysate and rSULT1A3 was similar (Figure 5C), which indicated that HEK-SULT1A3 cells have displayed the activity of SULT1A3 enzyme to catalyze sulfonation reaction.

### Effect of Chemical Inhibitor on Formononetin Sulfonation

**FIGURE 6:**
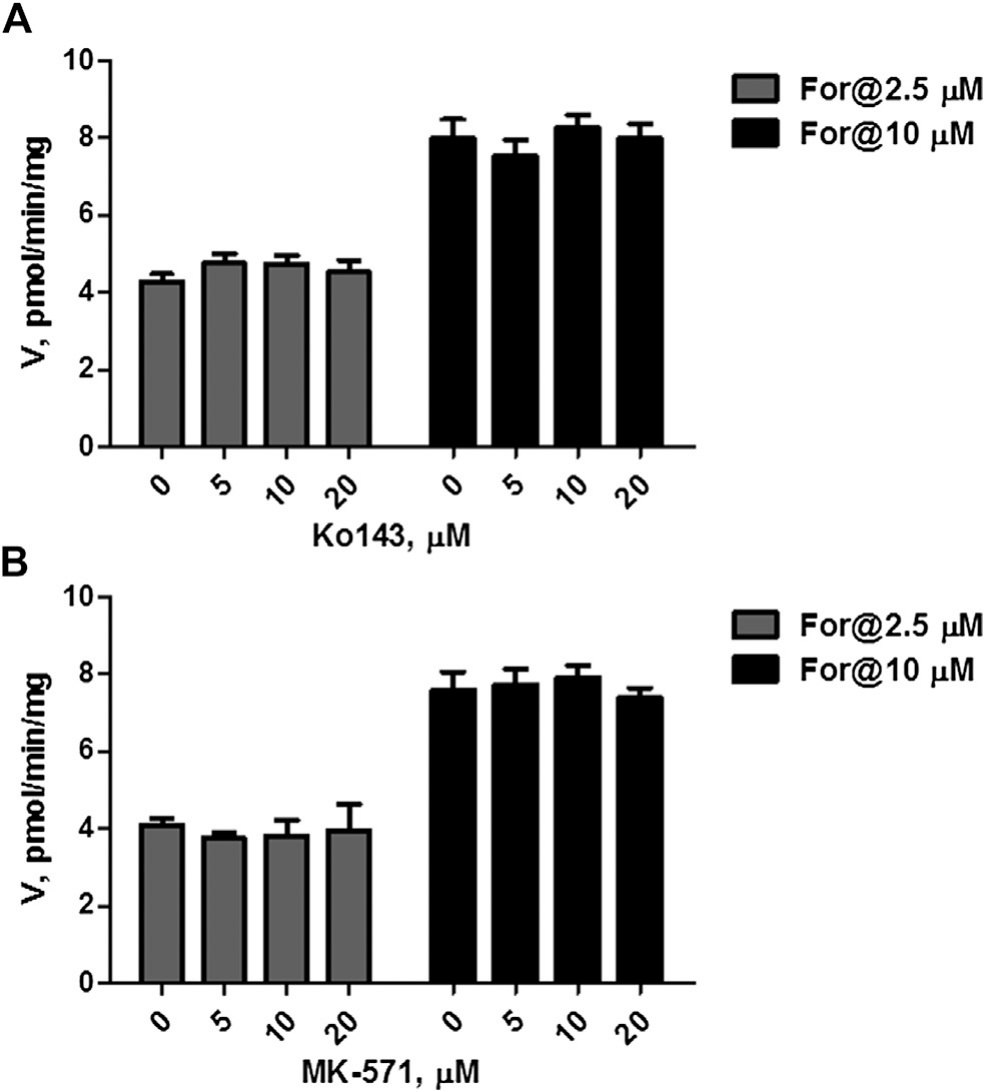
Effects of chemical inhibitors on formononetin (2.5 and 10 μM) sulfonation in HEK-SULT1A3 cell lysate. **(A)** Effects of Ko143 on formononetin sulfonation. **(B)** Effects of MK-571 on formononetin sulfonation. Each data point was shown as mean ± SD (n = 3).

The effects of Ko143 (a selective BCRP inhibitor) and MK-571 (a pan MRPs inhibitor) on formononetin (2.5 and 10 μM) sulfonation were evaluated using HEK-SULT1A3 cell lysate. Ko143, at the concentration of 5–20 μM, did not show any effects on formononetin sulfonation (Figure 6A, *p* > 0.05). Likewise, MK-571 at all tested concentrations (5–20 μM) did not alter the generation of F-O-S (Figure 6B, *p* > 0.05). These results indicated that sulfonation of formononetin mediated by cell lysate was not modulated by Ko143 and MK-571.

### Effect of Chemical Inhibitor on Sulfate Excretion

**FIGURE 7:**
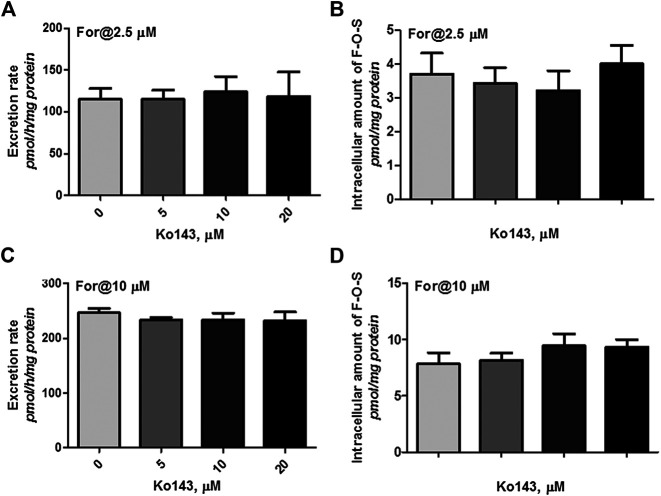
Effects of Ko143 (5–20 μM) on formononetin (2.5 and 10 μM) disposition in HEK-SULT1A3 cells. Effects of Ko143 on the excretion rates **(A)** and intracellular amounts **(B)** of formononetin sulfate at a low loading concentration of formononetin (2.5 μM). Effects of Ko143 on the excretion rates **(C)** and intracellular amounts **(D)** of formononetin sulfate at a high loading concentration of formononetin (10 μM). Each data point was shown as mean ± SD (n = 3).

Cellular expression of MRPs family transporters in wild type HKE293 and HEK-SULT1A3 cells has been detected using reverse transcription-polymerase chain reaction and western blotting method in our previous publications (Sun et al., 2015). The results indicated that BCRP and MRP4 were the dominant efflux transporters in HEK293 cells. And compared to wild type HEK293 cells, the expression level of BCRP and MRP4 was not changed in the engineered HEK-SULT1A3 cells. Furthermore, the role of BCRP and MRP4 involvement into formononetin sulfate excretion was determined using HEK-SULT1A3 cells. At a low loading concentration of formononetin (2.5 μM), Ko143 (5–20 μM) showed no or negligible effects on excretion rate and intracellular accumulation amount of F-O-S (Figure 7A,B, *p* > 0.05). Similarly, the sulfate excretion and intracellular accumulation were also not modulated by Ko143 (5–20 μM) when a high loading concentration (10 μM) of formononetin was used (Figure 7C,D, *p* > 0.05). These results suggested that BCRP was not involved in the excretion of formononetin sulfate in HEK-SULT1A3 cells.

**FIGURE 8:**
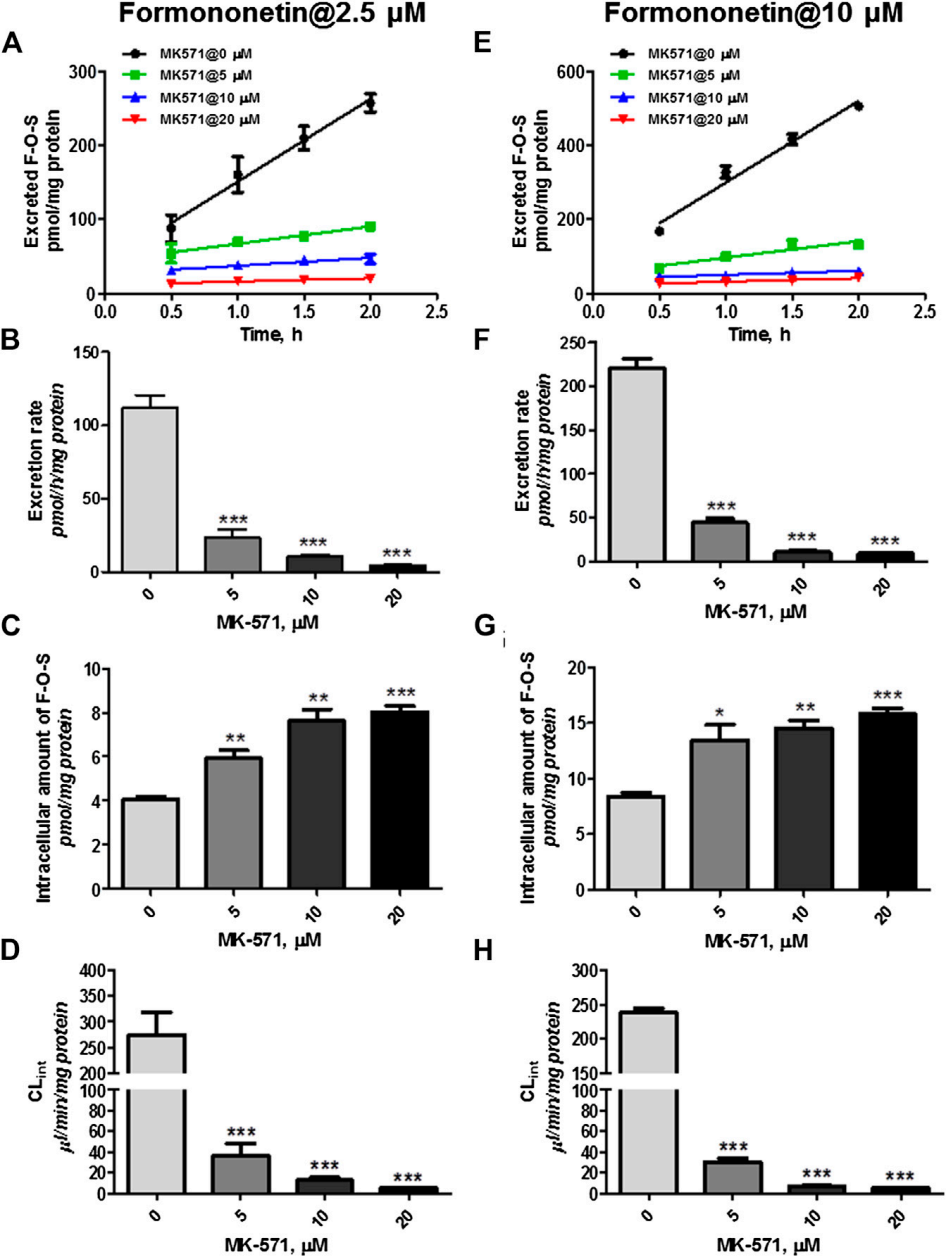
Effects of MK-571 (5–20 μM) on formononetin (2.5 and 10 μM) disposition in HEK-SULT1A3 cells. Effects of MK-571 on the excretion profile **(A)**, intracellular amounts **(B),** and efflux clearances **(C)** of formononetin sulfate at a low loading concentration of formononetin (2.5 μM). Effects of MK-571 on the excretion profile **(D)**, intracellular amounts **(E),** and efflux clearances **(F)** of formononetin sulfate at a high loading concentration of formononetin (10 μM). *p < 0.05, **p < 0.01, ***p < 0.001 compared with control. Each data point was shown as mean ± SD (n = 3).

However, coincubation of MK-571 (5–20 μM) and formononetin (dosing of 2.5 μM) resulted in substantial reduction (79.1%–94.6%, *p* < 0.001) in excretion of F-O-S (Figure 8A, B). On the contrary, MK-571 at higher concentrations (5–20 μM) caused an obvious elevation (145.6%–197.8%, *p* < 0.01 and *p* < 0.001) in intracellular amount of F-O-S (Figure 8C). It was not surprising that a remarkable decrease of CL_ef,app_ (85.3%–98.0%, *p* < 0.001) was observed in the presence of MK-571 at all tested concentrations (Figure 8D).

Similar effects of MK-571 on sulfate disposition were observed when a higher loading dose of formononetin (10 μM) was used. MK-571 (at the concentration of 5–20 μM) markedly decreased (79.8%–95.7%, *p* < 0.001) the excretion rates (Figure 8E,F) while it increased (160.6%–188.8%, *p* < 0.001) the intracellular level of F-O-S (Figure 8G). Further, CL_ef,app_ values of F-O-S were obviously reduced (87.3%–97.7%, *p* < 0.001, Figure 8H) under effect of MK-571. Taken together, the effect of MK-571 on formononetin sulfonation indicated that one or more MRPs family transporters played an important role in the excretion of formononetin sulfate.

### Effect of MRP4 Knockdown on Formononetin Sulfonation in HEK-SULT1A3 Cells

**FIGURE 9:**
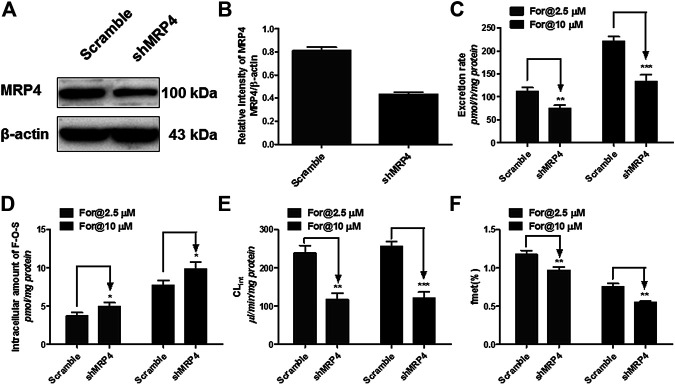
Effect of MRP4 silencing on formononetin (2.5 and 10 μM) disposition in HEK-SULT1A3 cells. **(A)** Western blot of MRP4 after transient transfection by selected shRNA. **(B)** Effects of gene silencing on the protein level of MRP4. **(C)** Effects of gene silencing on the excretion rates of formononetin sulfate. **(D)** Effects of gene silencing on the intracellular amounts of sulfate. **(E)** Effects of gene silencing on the excretion rates of sulfate. **(F)** Effects of gene silencing on the cellular sulfonation (f_met_) of formononetin. *p < 0.05, **p < 0.01, ***p < 0.001 compared with control. Each data point was shown as mean ± SD (n = 3).

MRP4 was the only expressed MRPs family transporter in HEK-SULT1A3 cells, and the effect of MRP4 on formononetin sulfonation transport was determined through gene silencing. The protein expression of MRP4 in HEK-SULT1A3 cells was significantly knocked down through transient transfection of selected shRNA (Figure 9A). Semiquantitative analysis of MRP4 was performed depending on band intensities, and *β*-actin was used as internal control. After transfection with shRNA, the protein expression of MRP4 was reduced by 44.7% (*p* < 0.01) (Figure 9B) in HKE-SULT1A3 cells. MRP4 knockdown led to obvious reductions (>32.8%, *p* < 0.01) in sulfate excretion rates (Figure 9C) but with significant elevations (>127.4%, *p* < 0.05) in intracellular amounts of F-O-S (Figure 9D) as expected. Accordingly, shMRP4 also caused obvious decrease (>50.6%, *p* < 0.01) in CL_ef,app_ values of F-O-S (Figure 9E). These results indicated that MRP4 was involved in excretion of formononetin sulfate. Furthermore, the role of MRP4 in formononetin cellular sulfonation was evaluated according to the f_met_ values. Interestingly, MRP4 silencing caused substantial decreases in cellular sulfonation of formononetin (17.3%–26.7%, *p* < 0.01) (Figure 9F).

## Discussion

Sulfonation is an important phase II metabolism mediated by sulfotransferases (Kauffman, 2004; Dubaisi et al., 2019) and regulates the disposition of numerous endo- and xenobiotics (James and Ambadapadi, 2013; Garbacz et al., 2017). Moreover, we and other researchers have discovered that phenolic compounds, especially the flavonoids, are susceptible to glucuronidation and sulfonation (two important metabolic pathways) (Chen et al., 2014; Najmanova et al., 2019; El Daibani et al., 2020). Due to the attention, the glucuronide metabolite of formononetin has been identified and quantified in rat plasma after oral administration (Wang et al., 2014; Shi et al., 2015), which indicated that glucuronidation may be an important clearance mechanism for formononetin *in vivo*. However, no sulfonation of formononetin has been reported, which means that the contribution of sulfonation to formononetin disposition may be ignored. Hence, it is necessary to evaluate the characterization of formononetin sulfonation. Furthermore, elucidation of the transporters responsible for excretion of formononetin sulfate will help promote understanding of pharmacokinetic behaviors of formononetin and its sulfate metabolites.

Oral bioavailability is a major factor in determining the biological actions of formononetin *in vivo*. Hence, it is critical to clarify the important factors involved in its oral bioavailability. As sulfonation was an important pathway for drugs clearance, characterization of formononetin sulfonation assumes great importance in understanding of its disposition and bioavailability. In this study, we for the first time identified that SULT1A3 played important role in formononetin sulfonation, because formononetin sulfonation was significantly correlated with SULT1A3 protein levels in a bank of individual human intestine S9 factions (Figure 4). It is well known that SULT1A3 is barely detected in human liver, but it is highly expressed in jejunum and intestine (Gamage et al., 2006). Thus, it was highly possible that intestinal sulfonation had impact on the oral bioavailability.

Further, the sulfonation of formononetin and its sulfate excretion were determined using SULT1A3 overexpressing HEK293 cells (HEK-SULT1A3). As expected, HKE-SULT1A3 cells catalyzed formononetin formation of one sulfate like the expressed human SULT1A3 enzyme. Sulfonation of formononetin followed the classical Michaelis-Menten kinetics (Figure 5). It was not surprising because SULT1A3 mediated sulfonation of flavonoids (e.g., chrysin, apigenin, and hesperetin) often showed Michaelis-Menten kinetics (Li et al., 2015; Sun et al., 2015). The V_max_ value of rSULT1A3 was much higher than cell lysate (Figure 5C), because the SULT1A3 enzyme was more concentrated in rSULT1A3 than in cell lysate. In addition, the K_m_ values of formononetin sulfonation derived from two types of enzyme materials were similar (*p* > 0.05). The results indicated that the rSULT1A3 enzyme and HEK-SULT1A3 cell lysate showed a high similarity in the sulfonation profile, providing strong evidence that HEK-SULT1A3 cells possessed a high conjugation activity toward formononetin owing to stable transfection of SULT1A3.

Furthermore, MRP4 was primarily responsible for the excretion of formononetin sulfate. These results were confirmed by the following strong evidence. First, the pan inhibitor of MRP family transporters (MK-571) at 20 μM almost completely suppressed (>94%) excretion of formononetin sulfate (Figure 8). Second, MK-571 showed no effects on formononetin sulfonation (Figure 6), which made it clear that alterations in sulfate excretion caused by MK-571 were mainly attributable to suppression of the functions of MRPs. Third, selective knockdown of MRP4 was followed by observable reduction in sulfate excretion rates (Figure 9).

Because BCRP was one of the major efflux transporters expressed in HEK-SULT1A3 cells (Sun et al., 2015), the contribution of BCRP to sulfate excretion was also determined. All results proved that contribution of BCRP to the excretion of formononetin sulfate was none or negligible. That is because when Ko143 (a potent and selective inhibitor of BCRP) was used, the excretion rates and intracellular levels of formononetin sulfate were not altered compared to the control (Figure 7). This was not surprising, as similar results have been obtained in our previous studies (Sun et al., 2015; Li et al., 2015; Liu T. et al., 2018). In addition, sulfonation of formononetin was not affected by Ko143 (Figure 6), which further convinced us that BCRP was not responsible for the efflux of formononetin sulfate. In a recent study, chrysin and chrysin-7-sulfate have shown stronger inhibitory effects on BCRP (Mohos et al., 2020). Therefore, we speculate that formononetin and/or its sulfate may also be an inhibitor of BCRP, which will prove why BCRP does not participate in the cellular excretion of formononetin sulfate. However, further evidence should be provided to support or disprove this hypothesis.

Ko143 and MK-571 have been widely used as the chemical inhibitor of BCRP and MRP4 *in vitro* and *in vivo* (Bertollotto et al., 2018; Drennen et al., 2018; Kanda et al., 2018; Feng et al., 2019). Hence, we have selected and used Ko143 and MK-571 to inhibit the transport activities of BCRP and MRPs in our study. Due to complicacies of chemical inhibitors in evaluation of sulfate excretion, the effects of Ko143 and MK-571 on formononetin sulfonation were determined. It was shown that both Ko143 and MK-571 did not alter sulfonation rates of formononetin mediated by HKE-SULT1A3 cell lysate (Figure 6). This result provided strong evidence that the reduction in sulfate excretion was mainly due to activities inhibition of transporters.

Our finding that human cells expressing SULT1A3 were active in metabolizing formononetin lent a strong support to notion that sulfonation plays an important role in disposition of formononetin. The sulfate metabolite of formononetin was also efficiently generated upon incubation of Caco-2 cells (Chen et al., 2005). In addition, significant amount of formononetin sulfate was found in intestine using mouse intestinal perfusion model (Jeong et al., 2005). Therefore, the role of sulfonation in determining the pharmacokinetics of formononetin might have been underestimated. Based on previous studies, flavonoids and/or their conjugate metabolites (glucuronide or sulfate) have shown significant interactions with metabolism enzymes and transporters (Li and Paxton, 2013; Mohos et al., 2020), raising the potential for interactions with conventional drug therapies. Furthermore, formononetin was identified as an inhibitor of CYP enzymes (Arora et al., 2015). However, the biological activities of formononetin sulfate remain underexplored. Due to the considerable sulfonation metabolism of formononetin, further studies should be devoted to the potential pharmacological effect of its sulfate and the impact of sulfate on the pharmacokinetics, efficacy, and toxicity of drugs.

HEK-SULT1A3 cells have been proved to be an appropriate model for evaluation of the cellular disposition processes of formononetin and its sulfate. That is because 1) formononetin sulfate did not need to be prepared as it could be generated during the experiments; and 2) formononetin has been reported to enter cells mainly through passive diffusion (Singh and Wahajuddin, 2011). Therefore, the uptake rates and intracellular amounts of formononetin when incubated with HEK-SULT1A3 cells have not been affected by chemical inhibitors (Ko143 and MRP4) and protein knockdown of efflux transporters. Furthermore, the rapid uptake of formononetin into HEK-SULT1A3 cells indicated that the sulfonation of formononetin by SULT1A3 in HEK-SULT1A3 cells will not be restricted by the intracellular amounts of parent compound. The results convinced us that efflux transporter MRP4 played an important role in the disposition of sulfate metabolite and formononetin.

We have shown for the first time that MRP4 silencing led to obvious reductions in cellular sulfonation (f_met_) of formononetin (Figure 9), which suggested that MRP4 potentially mediated the total sulfonation in cells. However, the values of f_met_ obtained at 10 μM were lower than that at 2.5 μM formononetin dose. That was not surprising, because f_met_ (%) was calculated by dividing the total amount of sulfate generated (including excreted and intracellular sulfate) by dosed formononetin. Although the amount of sulfate formed at 10 μM of formononetin was higher, when divided by the larger dosing, the calculated f_met_ (%) turned into lower than that at 2.5 μM of formononetin. It was noteworthy that the f_met_ (%) values showed a contradictory result with that in our previous publication (Liu et al., 2018). There was a possibility that the sulfonation rate of liquiritigenin was much higher than formononetin. Therefore, the amount ratio of liquiritigenin sulfate generated at 10 μM over at 2.5 μM in HEK-SULT1A3 cells was much higher than that of formononetin sulfate, which led to contradictory result in spite of the similar experimental conditions.

Although formononetin has shown satisfactory intestinal uptake (Singh and Wahajuddin, 2011; Almeida et al., 2015), the bioavailability of unchanged formononetin was found to be poor, approximately 3% (Singh and Wahajuddin, 2011). It was noted that glucuronide and/or sulfate of formononetin in circulating plasma were much higher than parent compound (Singh and Wahajuddin, 2011; Shi et al., 2015), which indicated that the poor bioavailability may be due to the extensive conjugation reaction (i.e., glucuronidation and/or sulfonation) *in vivo*. Hence, inhibition of the corresponding metabolism or activities of enzymes will contribute to the improvement of bioavailability (Izgelov et al., 2018; Najmanova et al., 2019; Ravisankar et al., 2019). In the present study, we have confirmed that inhibition of MRP4 activity or decreasing MRP4 expression led to a reduced formononetin sulfonation. Due to the extensive distribution of MRP4 in various tissues/organs, MRP4 should be a key factor for the elimination and distribution of formononetin sulfate, even the bioavailability of formononetin.

## Conclusion

Formononetin generated one sulfate metabolite after incubation with rSULT1A3 and HEK-SULT1A3 cell lysate, and the enzyme kinetics followed Michaelis-Menten model. HEK-SULT1A3 cells could mediate the formation and excretion of formononetin sulfate (F-O-S). Furthermore, MK-571 (the pan inhibitor of MRPs) at all test concentrations remarkably decreased the excretion rate and efflux clearance (CL_ef,app_) of formononetin sulfate, whereas Ko143 (the selected inhibitor of BCRP) showed no effect on sulfate excretion. Knockdown of MRP4 also caused substantial reduction in sulfate excretion. It was worth noting that MRP4 gene slicing further led to significant reduction in cellular sulfonation of formononetin. Taken together, MRP4 could effectively mediate the excretion of formononetin sulfate into extracellular media and played a regulation role in cellular sulfonation.

## Data Availability Statement

The datasets presented in this study can be found in online repositories. The names of the repository/repositories and accession number(s) can be found in the article/Supplementary Material.

## Authors Contributions

HS and FL participated in research design. HS, FL, SP, WL, XW, CL, RY, ZZ, and XY conducted experiments. DF and SX contributed new reagents or analytic tools. HS, FL, SP, and WL performed data analysis. HS wrote or contributed to the writing of the manuscript.

## Funding

This work was supported by the National Natural Science Foundation of China (81703801), Postdoctoral Research Foundation of China (2018M632764), Henan Province Young Talents Lifting Project (2019HYTP025), Postdoctoral Research grant in Henan Province (001801014), Innovative Research Team (in Science and Technology) in University of Henan Province (19IRTSTHN004), Key Project of Science and Technology Research Funded by the Educational Commission of Henan Province (18A350004), and Guangdong Provincial Key Laboratory of Drug Non-Clinical Evaluation and Research (2018B030323024).

## Conflict of Interest

The authors declare that the research was conducted in the absence of any commercial or financial relationships that could be construed as a potential conflict of interest.

## Abbreviations

HEK, human embryonic kidney; F-O-S, formononetin sulfate; Km, Michaelis-Menten constant; MRP, multidrug resistance-associated protein; PAPS, 3′-phosphoadenosine-5′-phosphosulfate; shRNA, short-hairpin RNA; SULT, Sulfotransferase; Vmax, maximal velocity.
